# Biodegradation of Phenol in Synthetic Wastewater Using a Fixed Bed Reactor With up Flow Sludge Blanket Filtration (FUSBF)

**DOI:** 10.5539/gjhs.v7n7p120

**Published:** 2015-03-26

**Authors:** Mohammad-Javad Ghannadzadeh, Ahmad Jonidi-Jafari, Abbas Rezaee, Reza Darvishi Cheshmeh Soltani

**Affiliations:** 1 Department of Environmental Health Engineering, Faculty of Medical Sciences, Tarbiat Modares University, Tehran, Iran; 2 Department of Environmental Health Engineering, School of Health, Arak University of Medical Sciences, Arak, Iran

**Keywords:** industrial wastewater, biological degradation, phenolic compounds, fixed bed USBF

## Abstract

In the present study, the removal of phenol from synthetic wastewater was evaluated in a fixed bed reactor with up flow sludge blanket filtration (FUSBF) in comparison with a typical USBF system. At hydraulic retention time (HRT) of 24 hours and solid retention time (SRT) of 20 day, the effect of initial concentration of phenol (260-1020 mg/L) on phenol and chemical oxygen demand (COD) removal efficiency (%) was investigated in both systems. The effect of the presence of fixed bed was determined throughout the operational period.

The results showed that the FUSBF system had a better ability than the typical USBF system in terms of phenol and COD removal. The average phenol and COD removal at phenol concentration of 312 mg/L was 97.52% and 92.82% for the FUSBF system and 92.80% and 82.18% for the typical USBF system, respectively. At HRT of 24 h and organic loading rate (OLR) of 30 g/m^-3^.h^-1^, the amount of phenol removal was 82.1%. At OLR of 30 g/m^-3^.h^-1^, role of fixed bed in phenol and COD removal was 25.01% and 29.3%, respectively, overall, the FUSBF system has a higher efficiency and ability than that of typical USBF and can be used for the purification of industrial wastewater containing refractory organic compounds such as phenol.

## 1. Introduction

Phenol and its compounds have many applications in chemical processes and are used as structural units in the synthesis of many organic compounds (Kirk-Othmer, 1998). They are applied in different industries such as petrochemical, oil refinery, insecticides, resin, plastic, pharmaceutical, leather, textile and paper. The concentration of phenolic compounds in the effluents of these industries is varied between one and hundreds of milligrams per liter (Pan et al., 2008; Quintanilla et al., 2008). Phenolic compounds are classified as mutagenic, teratogenic, and carcinogenic compounds (Quintanilla et al., 2008). The United States Environmental Protection Agency (USEPA) has introduced these compounds as the priority pollutants due to their inhibitory effect on biological systems (Chang et al., 1995; Rappoport, 2003; 2004, Kinsley & Nicell, 2000). At concentrations higher than 1 mg/L, phenol adversely affects the life of fishes and consequently threatens aqueous environments (Busca et al., 2008). Therefore, phenol-containing wastewater must be purified before discharge into the environment. Several physical, chemical, and biological methods were applied for phenol removal (Karthik et al., 2008); However, phenol removal by biological methods are preferred to physical and chemical methods, because they are less risky and less costly than physical and chemical techniques (Liu et al., 1996). Biodegradation of wastewater contaminants is varied and diverse. Anoxic and aerobic reactors are used for denitrification and removal of organic matters. In addition, hybrid anoxic and aerobic reactors are applied for pre-denitrification with a much more sludge return in sewages containing high amounts of organic matter (Melcer & Nutt, 1988). Hang (1985) studied wastewater treatment of diet soft drinks factories through anoxic-aerobic method and found that in addition to stability, the COD removal rate increases in this system.. Although recent studies have introduced an advanced system of fixed film reactor (Glass et al., 2008), the technology requires the formation of biofilm which *per three* needs a period for acclimatization of microbial mass in industrial wastewater containing high concentrations of pollutants (Roeselers et al., 2008; Tziotzios et al., 2005). Many studies regarding the types of suspended growth and fixed film systems have been performed for the evaluation of phenol removal; most of them have reported higher yields with fixed film than suspended growth systems (Wang et al., 2008). Although numerous studies have used aerobic, anoxic, and anaerobic methods to remove phenol from wastewater, no report was found about the application of up flow sludge blanket filtration (USBF) and fixed bed up flow sludge blanket filtration (FUSBF) for treating domestic and industrial wastewaters containing phenol. This approach can be used to reclaim domestic and industrial wastewaters and update the existing wastewater treatment plants (Rajakumar et al., 2011; Su et al., 2004). In this process, the wastewater enters the anoxic area and then enters from its bottom into an aeration basin; after overflow, it enters the sedimentation zone through the lower part of a clarifier. The remaining materials are returned from the bottom of the sedimentation (Su et al., 2004, Leva, 2001). Single-stage or two-stage design and operation can be carried out. All of these processes have been upgraded in a bioreactor resulting in a significant decrease in size and cost compared to other modified activated sludge systems (Punal et al., 2002, Kishida et al., 2006). The main aim of this investigation was to evaluate the capability of a single-stage FUSBF for removal of phenol from synthetic wastewater. In the following, the effect of hydraulic retention time (HRT) and organic loading rate (OLR) on phenol and COD removal was assessed.

## 2. Materials and Methods

### 2.1. Start-Up

**Figure 1:**
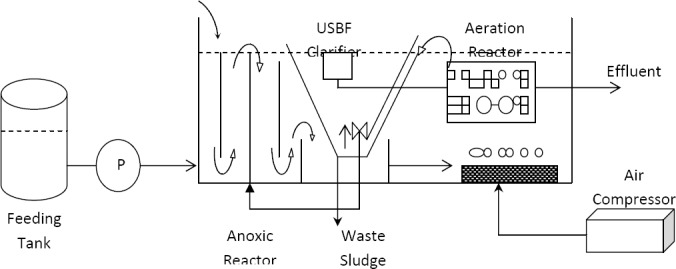
Schematic diagram of the lab-scale FUSBF integrated bioreactor

The experiments were carried out in two well-matched, equal-size hybrid bioreactors of FUSBF and USBF in lab-scale; they were rectangular plexiglass tanks with a total volume of 20 L (Figure 1).

For FUSBF hybrid bioreactor, an aerobic fixed film was installed as an appropriate unit for further biological removal in the aerobic zone. Twenty percent of the aerobic reactor volume was filled by a media made of high density polyethylene (HDPE). Support material surface area, porosity, mean diameter and density were 400 m^2^/m^3^, 56%, 14 mm and 97.0–92.0 g/cm^3^, respectively. Sludge from the clarifier of FUSBF directly turned back into the anoxic area. The return activated sludge (RAS) from clarifier was measured and controlled through the amount and time of sludge return. The amount of RAS to the anoxic area was 3 times of the average inflow. The reactors were operated at room temperature (25 °C). The required air for aeration was mixed in the aerobic zone by an air compressor with a maximum output of 90 L/min along with three air stone diffusers. Air flow was adjusted by hand valves so that the concentration of dissolved oxygen in the aerobic zone liquid was 4.0±0.5 and oxidation-reduction potential (ORP) in the anoxic area was about –30±40 mV. The pH of solution was maintained neutral throughout the experiments.

### 2.2 Operation Method

Synthetic wastewater, containing phenol as the carbon source, NO_3_NH_4_ as the nitrogen (N) source, and KH_2_PO_4_ and K_2_HPO_4_ as the source of phosphate (P), entered into the system throughout the operation and minor elements for biomass were added into the synthetic wastewater as follows: FeCl_3_.6H_2_O: 1.5 g/L, H_3_BO_3_: 0.15 g/L, CuSO_4_.5H_2_O: 0.03 g/L, KI: 0.03 g/L, MnCl_2_.4H_2_O: 0.12 g/L, NaMnO_4_.2H_2_O: 0.06 g/L, ZnSO_4_.H_2_O: 0.12 g/L and CoCl_2_.6H_2_O: 0.15 g/L (31). The composition of wastewater was determined based on the ratio of COD/N/P: 100/5/1. The required sludge was obtained as seed from a municipal sewage treatment plant in Arak. The operational flow rate for both FUSBF and USBF bioreactors were 10-60 L/dThe operational parameters for both FUSBF and USBF bioreactors are presented in Table 1.

**Table 1:** Operational parameters for the lab-scale FUSBF and USBF bioreactors.

Parameter	Anoxic zone	Aerobic	Clarifier
**Volume (L)**	6	9	2
**HRT (h)**	2.4-14.4	3.6-21.6	0.8-4.8
**SRT (day)**		20	
**Average MLSS (mg/L)**	3290	3735	6200
**Average MLVSS (mg/L)**	2575	2865	

Phenol was added increasingly and stepwise (from 0 to 260 mg/L) to the FUSBF and USBF bioreactors during the startup. Phenol was the sole carbon source for growth of microorganisms and formation of biofilm. Synthetic wastewater was continuously prepared and supplied into both FUSBF and USBF reactors and was pumped with a flow of 20 L/day under the mentioned operational conditions. During the startup period, this flow level was used for 3.7 h and 10.8 h in anoxic and aerobic zones, respectively, at a HRT of 24 hours and a SRT of 20 days. Microorganisms and the biofilm were well grown under control in different concentrations of pollutants. Treatment of inflow began when the biofilm was formed and the pollutants concentration in the bioreactors outflow became constant. Under these conditions, it takes 165 and 172 days to startup the developed FUSBF and typical USBF bioreactors, respectively. Then the efficiency of the systems was evaluated with different initial concentrations of phenol and different HRT from 12 to 48 hours. The conditions for implementation of the stages were determined using a combination of parameters given in Table 1. At each stage, the experiments were performed when the bioreactors attained steady-state conditions.

### 2.3 Sampling and Analysis

Using synthetic wastewater, the systems reached to stable conditions and then were utilized. After seeding, the biofilm formed in aerobic reactor of the developed FUSBF bioreactor was visualized (in terms of morphology) using scanning electron microscopy (SEM). For this, the media was randomly sampled from various parts of the aerobic reactor at the end of the experiment in the stable stage. The withdrawn media were dried in darkness at room temperature for 12 hours before observation (Trinet et al., 1991). The biofilm concentration formed on the moving bed was determined according to the procedure used by Darvish (Soltani et al., 2013). The samples were collected from the reactor at each operational run. Parameters measured included the levels of phenol, dissolved oxygen (DO), PH, (ORP), COD, mixed-liquor suspended solid (MLSS), and mixed-liquor volatile suspended solid MLVSS. Samples were collected and analyzed from inflow and outflow of the anoxic and aerobic reactors, as well as from clarifier. Temperature, DO, ORP, and PH were measured every day, immediately prior to sampling. To remove particles, the samples were passed from a 0.45 micron filter. MLSS, MLVSS and COD were analyzed according to standard methods for the examination of water and wastewater (APHA, 2005). Suitable probes were used to measure ORP, DO, and PH. Phenol concentration was measured using high performance liquid chromatography (HPLC); to this end, 20 mL of sample was injected into a 4.0-250 mm ODS C18 column (specifications). A mixture of acetonitrile/deionized water/acetic acid (50/50/1.5 v/v/v) as the mobile phase solution was used at a flow rate of 2 mL/min. The samples were then analyzed at 270 nm using the UV-visible detector.

## 3. Results and Discussion

### 3.1 Startup of Reactor, Acclimatization of Biomass, and Formation of Biofilm

During acclimatization of the liquid mixture, the initial concentration of phenol was gradually increased from 60 to 260 mg/L. During this period, increase in phenol degradation at each stage of phenol initial input was considered as the fulfilling of acclimatization. Then the next stage of phenol increase started. In other words, increased rate of phenol biodegradation in a range of increasing phenol concentrations confirmed that acclimatization was fulfilled, the microbial population boosted and propagated, and biomass gained the capacity and ability to degrade phenol under the conditions existing in both bioreactors of USBF and developed FUSBF. So biomass was prepared as a suitable seed for utilization and operation of bioreactors. The total time required for the acclimatization stage for both USBF and developed FUSBF bioreactors was 172 and 165 days, respectively. The total amount of inoculated biomass in the anoxic and aerobic reactors at startup in the USBF reactor was almost 2100 and 2480 mg/l, respectively.

### 3.2 Biomass Characterization of Biofilm Formation

**Figure 2:**
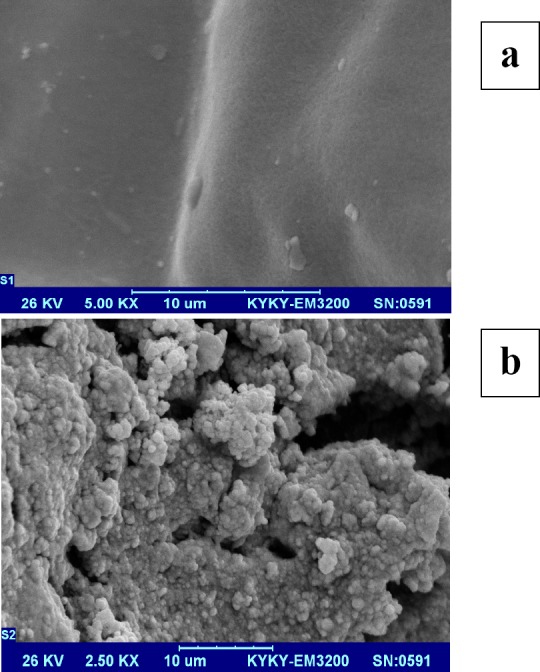
SEM images of (A) clean and (B) biofilm containing media

**Figure 3:**
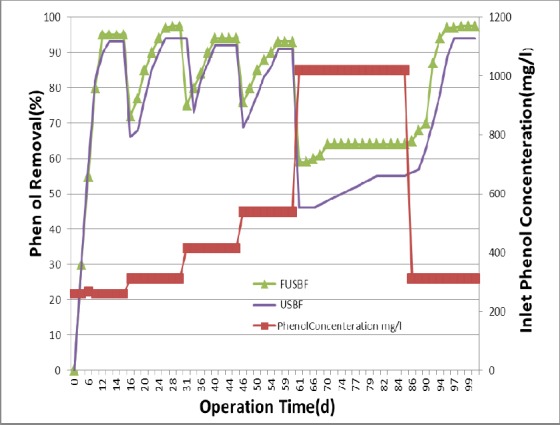
Phenol removal efficiency as a function of initial concentration of phenol ranging from 260 to1020 mg/L

SEM image of the initial clean media and the biofilm containing media was used to view and characterize the surface of fixed film medium in the aerobic FUSBF reactor. As shown in Figure 2, formation of biofilm in the fixed film aerobic reactor after seeding was confirmed by the results of SEM analysis. The formation of biofilm in the FUSBF is attributed to the hydrodynamic role that results from fix bed the media in the suspension (G´omez et al., 2003). The presence of a dense biofilm with a rough and cloven surface can be clearly seen in the SEM image (Figure 3).

The results showed that the media used was suitable for biofilm formation or formation of biofilm and no pre-seeding was required. Figure 2(a) shows the clean and uncovered biofilm and media. As can be seen, the rough surface of the existing media is an ideal surface for formation of biofilm. Figure 2(b) shows the growth and development of biofilm after biofilm formation in the aeration zone of the FUSBF system. The formation of a non-uniform dense biofilm with porous properties is clearly observable. Density and morphology of biofilm in the FUSBF bioreactor is an incentive to work on the concentration of phenol and oxygen and the ability to remove pollutants (Wang et al., 2005). The morphology of biofilm formation on fixed film media can be affected by the amount of media, cross-section, and slots and grooves of the media segments in the reactor (Maran et al., 2008).

### 3.3 Effect of Initial Concentration of Phenol on Its Removal Efficiency

After a successful startup and achieving a stable stage (less than 5% change in phenol output in a week at phenol concentration of 260 mg/L) and confirmation of biofilm formation via SEM, the reactors were operated under conditions of HRT=24 h and SRT=20 d for evaluation of phenol removal at different input concentrations of 312, 416, 520, 780, and 1020 mg/L. Experiments were performed when the conditions were stable; these stable conditions were the same for all experiments and had no statistically significant difference (less than 5%) for about 5-7 days between the results of experiments. The behavior of typical USBF and FUSBF bioreactors at HRT=24 h and SRT=20 d was evaluated at different initial concentrations of phenol. The removal of phenol and COD was displayed as a function of initial concentration of phenol (Figure 3). The removal efficiency in both USBF and FUSBF bioreactors can be seen. As shown in Figure 3, the amount of phenol removal in both USBF and FUSBF bioreactors rises and finally reaches to 92 and 97.5, respectively, and then remained constant for the next six days.

This increase in the efficiency of daily removal of 312 mg/L phenol during the operation shows that the seed inoculated or injected into the USBF and FUSBF bioreactors was able to degrade high concentration of phenol. In a similar approach, phenol concentration was increased in a stepwise manner to 416, 572, 728, and 1020 mg/L and both bioreactors were operated and evaluated until achieving steady-state condition. As seen, after a sharp increase in the concentration, for example from 416 mg/L to 572 mg/L and at any other concentration increments, the removal rate decreased at both reactors immediately the next day, although the rate of removal decline in the FUSBF reactor was less than the USBF reactor after each step of increase in concentration. For example, immediately after an increase in the concentration of phenol to 572 mg/L, the removal rate of the FUSBF reactor reached to 72.3% the next day, while declined to 63.5% in the USBF reactor. However, the removal rate increased again in both bioreactors in the next few days of operation. In general, by increasing the concentration of phenol, the removal process had a descending trend in both bioreactors, although the highest removal rate of decline was observed at 1020 mg/L in both reactors. At this concentration, the decrease in removal efficiency was higher in the typical USBF reactor than the FUSBF reactor. In fact, although the removal rate increased again after its sudden decline at the day next to sudden increase in phenol concentration to 1020 mg/L, and although both bioreactors were restored again and achieved steady state conditions, the time required to reach the steady state conditions was 20 days for FUSBF and 35 days for typical USBF bioreactor.

To confirm the capabilities of both FUSBF and typical USBF systems under the minimum inhibitory concentration of phenol and to test the revival behavior of the system caused by excessive load entered, the input concentration of phenol was reduced in a stepwise manner from 1020 mg/L, to 728, 572, 468, and 312 mg/L. At each concentration, both bioreactors achieved the stable conditions of removal. Figure 3 shows that by returning to phenol concentration of 312 mg/L, the removal efficiency in both bioreactors quickly reached to 90% of phenol removal. This high removal rate confirms that the inhibitory effect, occurred due to an increase in previous concentrations, was disappeared after decreasing the concentration (Rivas et al., 2013). Finally, phenol concentration was returned to 312 mg/L resulting in phenol degradation higher than 90% in both bioreactors. The above results clearly show that in the same operational conditions, the FUSBF bioreactor had higher resistance to the input phenol concentration than the USBF bioreactor, in particular at higher concentrations. In terms of the time, the FUSBF bioreactor restored and was able to accept higher concentrations in a shorter period. This higher resistance and ability to better revive at higher concentrations can be attributed to the protective reactions of extracellular polymeric substances which hold together the biofilm (Fang et al., 1996). In summary, the efficiency of phenol removal in steady-state conditions for input concentrations were 97.5% for FUSBF and 92.18% for typical USBF.

### 3.4 Effect of Phenol Concentration on the Removal of COD

**Figure 4:**
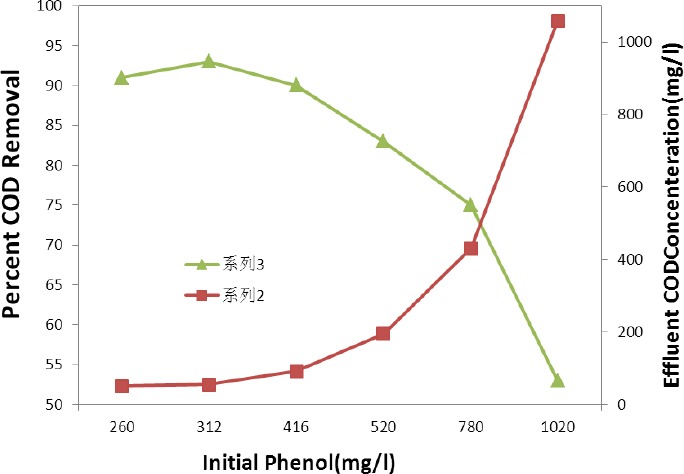
Variations of effluent COD concentration and COD removal efficiency as a function of initial phenol concentration

Changes in effluent COD concentration and COD removal along with initial phenol concentration are shown in Figure 4. COD removal efficiency was higher than 90% and effluent COD concentration was less than 100 mg/L for all concentrations of less than 416 mg/L, because more than 90% of phenol degraded in almost all cases of lower than 416 mg/L, which had no significant inhibitory effect on COD removal. But when the phenol concentration increased to 870 mg/L, the COD removal efficiency was reduced to 75%. As a result, the effluent COD concentration reached to 431 mg/L. This can be due to increased concentrations of phenol which may prevent the COD removal, as phenol concentrations of more than 416 mg/L declined the biodegradation of phenol (39). The COD concentration in the refined effluent in both bioreactors was higher than phenol; this can be attributed to the presence of COD arisen from intermediate compounds which degradation efficiency is generally lower than that of phenol (Sa et al., 2001).

### 3.5 The Effect of Phenol Concentration on SVI

**Figure 5:**
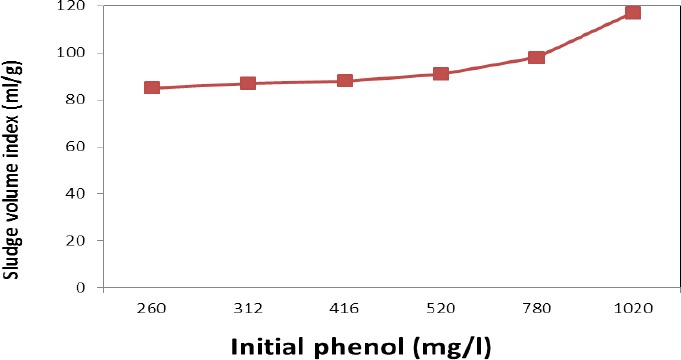
Variations of sludge volume index (SVI) with initial phenol concentration

Figure 5 depicts the changes in SVI as a result of the change in phenol concentration. For all concentrations lower than 416 mg/L, SVI was almost constant at about 85 ml/g. However, SVI increased at phenol concentrations higher than 416 mg/L and reached to 115 mg/L at phenol concentration of 1020 mg/L. Therefore, activated sludge microorganisms probably had no important inhibitory or conflicting effect when phenol concentration increased up to 416 mg/L. Overall, there were good deposition properties without any decay or inactivation. But SVI increased in concentrations higher than 416 mg/L due to inactivation and decay of microorganisms, resulting in unacceptable deposition characteristics.

### 3.6 The Effect of HRT on Phenol Removal

**Figure 6:**
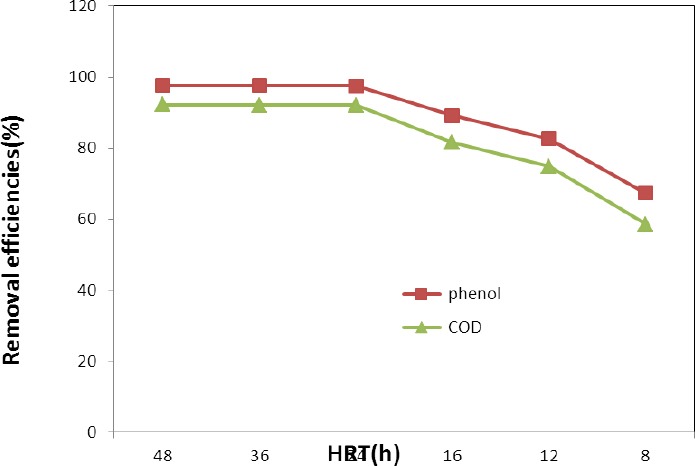
Variations of phenol and COD removal efficiencies under different HRTs at an initial phenol concentration of 312 mg/L

Biological wastewater treatment processes are cost effective only when they act highly efficient in a short time, otherwise the facilities size may not meet the requirements. Removal of phenol was evaluated at HRTs of 48, 36, 24, 12, and 8 hours in FUSBF under steady-state conditions. The phenol and COD removal under different HRTs are shown in Figure 6. The reactors were operated for a period of 20 days. As results, the phenol removal (%) for HRTs of 48, 36, 24, 18, 12 and 8 were 97.50, 97.38, 97.22, 89.12, 82.54, 67.34%, respectively. The removal of COD was higher than 81% for HRTs 48, 36, 24, and 18 hours, while at HRT of 12 hours, the removal efficiency was reduced to 74.8 4%.

### 3.7 Role of Media in FUSBF Performance

**Figure 7:**
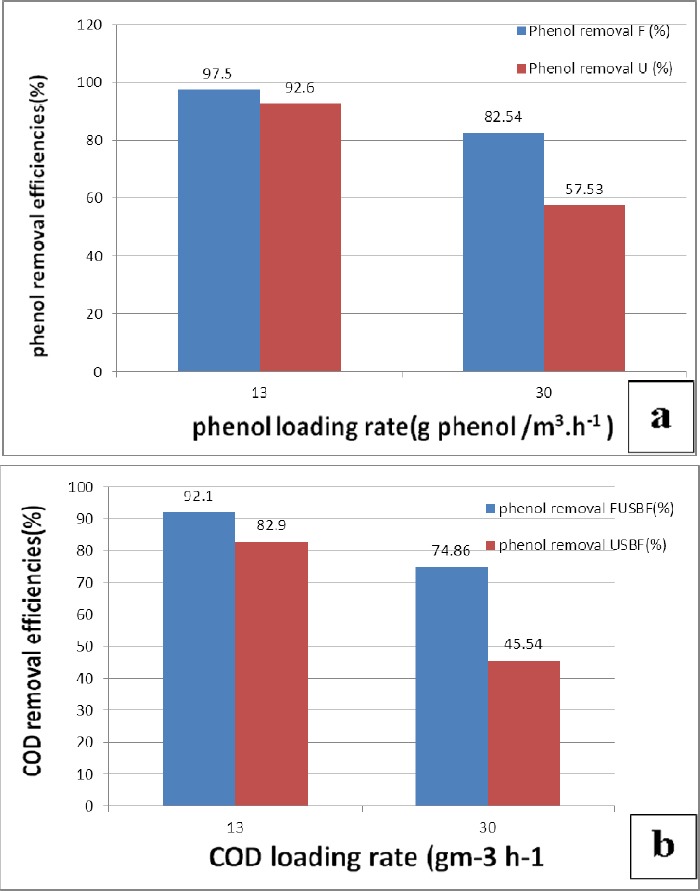
Average contribution of the biofilm in the efficiency of FUSBF for the removal of (a) phenol and (b) COD at different organic loading rates

To better understand and explain the role of fixed bed media in operation of FUSBF system, two different loads of phenol (13 and 30 g phenol/m^3^.h^-1^) were used and compared in both FUSBF and typical USBF. The average phenol and COD removal efficiencies are shown in Figure 7a, b, respectively.

The mean phenol and COD removal efficiency is shown in Figure 7b. As shown in Figure 7a, the role of fixed film media in the removal of phenol increases 4.9 % in the load 13g phenol/m^3^.h^-1^, while it increases 25.01% in the load 30g phenol/m^3^.h^-1^.

This increased share following increase in the load (concentration) may be due to consumption and attraction of more phenol load by the fixed film biofilm that result in lowering of the inhibitory effect of phenol on biomass in the reactor and delaying of the inhibitory effect by the fixed film biofilm. The role of fixed film media was greater in COD removal than in phenol removal. The role of fixed film media for COD removal of phenol loads of 13 and 30 g phenol/m^3^.h^-1^ was 9.2% and 29.3%, respectively, which represents an important effect of fixed film biofilm altogether on the entire process of system exploitation. This may be due to the presence and activity of fixed film biofilm in the reactor and thus an increase in the amount of mass conversion of substrate in the mixture liquid to convert biomass and form biofilm. In general, the efficiency of FUSBF for treatment of inhibitors with high concentrations such as phenol is promising

## 4. Conclusion

In the present work, an FUSBF process was developed and studied removal. The fix film USBF intergraded bioreactor at the optimum condition can be an effective technology for phenol removal from synthetic wastewater.

In this type of system microbial fixation and biofilm formation the support surface are two of the most important factors. Since they affect the levels of elimination of every pollution concentration in a synthetic wastewater. The results reveal that the FUSBF provides inproved phenol and COD removal efficiencies better than the conventional USBF, when it is operated as a single reactor with 20%media volume. The inhibition concentration of phenol in FUSBF and USBF are 1020 mg/l. The optimum HRT for the FUSBF is 24 h, at which the removal efficiencies of phenol and COD are greater than90% The critical phenol leading rate for the FUSBF is 30 g phenol/m-3.h. Which gives a phenol removal efficiency of 82.54(%) The reactor FUSBF demonstrates a high capacity for phenol removal compared to the USBF reactor. Accordingly the FUSBF is an efficient reactor phenol compound removal.
